# Inhibition of cdk9 during Herpes Simplex Virus 1 Infection Impedes Viral Transcription

**DOI:** 10.1371/journal.pone.0079007

**Published:** 2013-10-18

**Authors:** Mark Ou, Rozanne M. Sandri-Goldin

**Affiliations:** Department of Microbiology and Molecular Genetics, University of California, Irvine, California, United States of America; Dartmouth Medical School, United States of America

## Abstract

During herpes simplex virus 1 (HSV-1) infection there is a loss of the serine-2 phosphorylated form of RNA polymerase II (RNAP II) found in elongation complexes. This occurs in part because RNAP II undergoes ubiquitination and proteasomal degradation during times of highly active viral transcription, which may result from stalled elongating complexes. In addition, a viral protein, ICP22, was reported to trigger a loss of serine-2 RNAP II. These findings have led to some speculation that the serine-2 phosphorylated form of RNAP II may not be required for HSV-1 transcription, although this form is required for cellular transcription elongation and RNA processing. Cellular kinase cdk9 phosphorylates serine-2 in the C-terminal domain (CTD) of RNAP II. To determine if serine-2 phosphorylated RNAP II is required for HSV-1 transcription, we inhibited cdk9 during HSV-1 infection and measured viral gene expression. Inhibition was achieved by adding cdk9 inhibitors 5,6-dichlorobenzimidazone-1-β-D-ribofuranoside (DRB) or flavopiridol (FVP) or by expression of a dominant–negative cdk9 or HEXIM1, which in conjunction with 7SK snRNA inhibits cdk9 in complex with cyclin 1. Here we report that inhibition of cdk9 resulted in decreased viral yields and levels of late proteins, poor formation of viral transcription-replication compartments, reduced levels of poly(A)+ mRNA and decreased RNA synthesis as measured by uptake of 5-bromouridine into nascent RNA. Importantly, a global reduction in viral mRNAs was seen as determined by microarray analysis. We conclude that serine-2 phosphorylation of the CTD of RNAP II is required for HSV-1 transcription.

## Introduction

The largest subunit of RNA polymerase II (RNAP II) in eukaryotes contains a highly conserved C-terminal domain that consists of tandem repeats of the heptapeptide YSPTSPS, which is repeated 52 times in humans. Serine residues at positions 2 and 5 are reversibly phosphorylated during transcription [1]. While unphosphorylated RNAP II is recruited to promoters, after assembly of the pre-initiation complex, serine-5 becomes phosphorylated during initiation, primarily by the kinase cdk7, which is associated with the general transcription factor TFIIH [1,2]. Capping of the 5’ end of the nascent RNA is associated with initiation and serine-5 phosphorylation [1–4]. Transition into the elongation phase of RNAP II transcription requires phosphorylation of serine-2 by the kinase cdk9, which acts in conjunction with cyclin 1 in mammalian cells and the complex is referred to as P-TEFb for positive transcription elongation factor [2,3]. Following initiation, transcription is paused by the repressors DSIF and the negative elongation factor, NELF resulting in short transcripts that require the recruitment of cdk9 [3–5]. DSIF and NELF are phosphorylated by cdk9, relieving the transcriptional pause and cdk9 also then phosphorylates serine-2 of the CTD of RNAP II [3,6–13]. Phosphorylation of CTD serine-2 has also been shown to be required for co-transcriptional mRNA processing including splicing and polyadenylation [2,14–18]. 

During herpes simplex virus 1 (HSV-1) infection, it has been reported that RNAP II phosphorylation patterns are altered compared to uninfected cells, resulting in an intermediate form of RNAP II that migrates more slowly than the hypophosphorylated form but faster than the hyperphosphorylated form [19]. It was subsequently shown that the viral immediate early protein ICP22 and a viral kinase U_L_13 are required for this intermediate form of RNAP II [20,21]. The actual CTD phosphorylation sites for U_L_13 have not been identified, nor has the role that this intermediately phosphorylated form plays during viral infection been elucidated. It has also been shown that ICP22 associates with cdk9 and colocalizes with cdk9 and RNAP II [22,23]. Paradoxically, HSV-1 infection leads to a loss of RNAP II CTD phosphoserine-2 [24–26]. This occurs during times of highly active transcription of early and late genes during infection and in fact, there is a measurable decrease in total RNAP II levels at later times of HSV-1 infection [24,27]. We showed that this resulted from proteasomal degradation of RNAP II and could be prevented using proteasome inhibitors MG132 or lactacystin or the transcription elongation inhibitor actinomycin D [24]. We postulated that because the HSV-1 genome is transcribed from both DNA strands and it contains several regions where transcripts from different genes overlap, during highly active viral transcription, RNAP II elongating complexes might collide or pile up resulting in stalled complexes. Proteasomal degradation of stalled complexes would allow re-initiation and elongation through the former site of the stalled complex. Loss of serine-2 phosphorylation has also been shown to occur in cells that were transfected with a plasmid expressing HSV-1 protein ICP22, and this did not require any other viral factors or viral transcription [26]. Because it was shown that ICP22 binds cdk9 and that both can be found colocalized with RNAP II [23], it is possible that ICP22 may modulate cdk9 activity in some manner although how this might occur has not been demonstrated. 

Because of the decrease in phosphoserine-2 during HSV-1 infection, it has been proposed that serine-2 phosphorylated RNAP II may not be required for HSV-1 transcription elongation. To determine whether serine-2 phosphorylated RNAP II is required during HSV-1 replication, we inhibited cdk9 and observed decreased viral yields and reduced viral transcription, indicating that serine-2 phosphorylation of RNAP II CTD is required during HSV-1 replication. 

## Materials and Methods

### Cells, viral strains and plasmids

HeLa cells were grown on minimal essential medium (MEM) containing 10% new born calf serum. Rabbit skin fibroblasts (RSF) and Vero cells were grown on minimal essential medium supplemented with 8% fetal calf serum and 4% donor calf serum. HSV-1 wild-type (WT) strain KOS was described previously [28]. ICP27 null mutant 27-GFP encodes green fluorescent protein (GFP) in place of ICP27 coding sequences and has been described previously [29,30]. Mammalian expression vector pRc/CMV-dnCDK9-HA encoding a kinase-dead cdk9 was generously provided by Dr. Xavier Graña [31]. Plasmid pCMV2-FLAG-HEXIM1 was a generous gift from Dr. Qiang Zhou and it has been described previously [32]. 

### Virus infection and transfection

HeLa cells were infected with WT HSV-1 KOS or 27-GFP as indicated at a multiplicity of infection (MOI) of 10 and were incubated at 37°C for the times indicated in the figure legends. For transfection/infection experiments, plasmid DNA was transfected into cells using Lipofectamine 2000 reagent (Invitrogen) according to the manufacturer’s protocol. Cells were infected 24 h after transfection.

### Cell viability assay and single cycle viral growth curve analysis

HeLa cells seeded on 6-well tissue culture dishes were either mock infected or infected with HSV-1 KOS at an MOI of 1.0 for up to 16 hours as indicated in the figure legends. Cells were either incubated in MEM or MEM containing 100 µM 5.6-dichloro-1-β-D-ribofuranosyle-1H-benzimidazole (DRB) or 450 nM flavopiridol (FVP). For cell viability assays, cells were harvested at 4 h intervals and stained with the vital dye trypan blue solution (Sigma), followed by cell counting using a hemocytometer. For single cycle viral growth assays, cells were infected with HSV-1 KOS at an MOI of 1 in the presence or absence of DRB or FVP as indicated and were harvested at 4, 8, 12, and 16 h after infection. Virus titers were measured by plaque assays on Vero cells. The experiments were performed in triplicate. 

### Western blot analysis

HeLa cells were infected or transfected and then infected as indicated in the figure legends. At the times indicated, cells were washed with cold phosphate-buffered saline (PBS) and harvested in 2X ESS loading buffer (20 mM Tris, 5 mM EDTA, 4% SDS, 10% 2-mercaptoethanol, 20% glycerol), as described previously [29]. Cell lysates were fractionated on 5-15% gradient sodium dodecyl sulfate polyacrylamide gels and transferred to nitrocellulose membranes. Membranes were probed as described previously [29,33]. Primary antibodies used for immuno-blotting were as follows: rabbit polyclonal anti-Rpb1 N20 (Santa Cruz Biotechnology, Inc.) at 1:1,000; mouse monoclonal anti-RNAP II CTD phosphoserine-2 H5 (Abcam) at 1:2,500; mouse monoclonal anti-RNAP II CTD phosphoserine-5 H14 (Abcam) at 1:2,500; rabbit monoclonal anti-CDK9 C12F7 (Cell Signaling Technology) at 1:2,000; rabbit polyclonal anti-HEXIM1 ChIP grade (Abcam) at 1:1,000; mouse monoclonal anti-HA epitope HA-7 (Sigma-Aldrich) at 1:250; mouse monoclonal anti-FLAG epitope M2 (Sigma-Aldrich) at 1:1,000; rabbit polyclonal anti-Lamin A/C (Cell Signaling Technology) at 1:2,000; rabbit monoclonal anti-YY1 (Abcam) at 1:500; mouse monoclonal anti-ICP27 (P1119; Virusys) at a dilution of 1:5,000, mouse monoclonal anti-ICP4 (P1101; Virusys) at 1:5,000, mouse monoclonal anti-glycoprotein D (gD) (P1103;Virusys Corporation) at 1:5,000 and mouse monoclonal anti-glycoprotein B (gB) (P1123; Virusys Corporation) at 1:5,000 

### Immunofluorescence microscopy

RSF cells grown on glass cover slips were infected or transfected as described in the figure legends. Cells were either untreated or were incubated with MEM supplemented with DRB or FVP as indicated in the figure legends. Cells were fixed with 3.7% formaldehyde at the times indicated and immunofluorescent staining was performed as described previously [24,27,34]. Cells were stained with anti-ICP4 (P1101) at 1:500; anti-ICP27 (P1119) at 1:500; anti-RNAP II antibody ARNA3 (Research Diagnostics) at 1:50; anti-bromodeoxyuridine Ab-3 (Calbiochem) at 1:100; anti-HA (Sigma) at 1:500 and anti-FLAG (Sigma) at 1:500. 

### Bromouridine labeling and in situ hybridization

To label nascent, newly synthesized RNA, 4 mM 5-Bromouridine (BrU-Sigma-Aldrich) was added to the culture medium for 30 min at 37°C before fixation of the cells, which were subsequently stained with anti-BrU antibody (Ab3; Calbiochem). For poly(A)+ RNA hybridization, cells grown on coverslips in 24 well dishes were infected as described in the figure legends and were fixed with 3.7% formaldehyde and then overlaid with 70% ethanol at 4°C. Cells were rehydrated for 5 min at room temperature in 15% dimethylformamide in 2X SSC (1X SSC is 0.15 M NaCl plus 0.015 M sodium citrate) and then overlaid with 40 µl hybridization solution (15% formamide, 10% dextran sulfate, 40 µg yeast tRNA, 0.02% bovine serum albumin, 40 ng biotinylated oligo[dT] (Promega), RNAsin, 0.5 M dithiothreitol, 2X SSC) for 2 h at 37°C. Cells were washed twice with wash solution (15% formamide, 2X SSC and 0.1% NP-40) for 30 min at 37°C. Images were captured using a Zeiss Axiovert S100 microscope at a magnification of 100X. 

### Microarray analysis

HeLa cell monolayers were infected with WT HSV-1 KOS at an MOI of 10 for 8 hours. At 3 h after infection, cell culture medium was replaced with medium without inhibitors or medium containing 100 µM DRB or 450 nM FVP and incubation was continued for an additional 5 h. Total RNA was isolated from whole cell lysates with TrIzol reagent (Invitrogen). Poly(A)+ RNA was selected using an Oligotex mRNA mini kit (QIAGEN) according to the manufacturer’s protocol. Synthesis of cDNA and subsequent hybridization to custom arrays of HSV-1 transcript-specific probes was performed as previously described [35,36]. Statistical analysis was performed using a one-tailed t-test assuming unequal variance, comparing drug treated samples to corresponding control samples, with a 97.5% confidence limit. 

## Results

### RNAP II levels are reduced during HSV-1 infection

**Figure 1:**
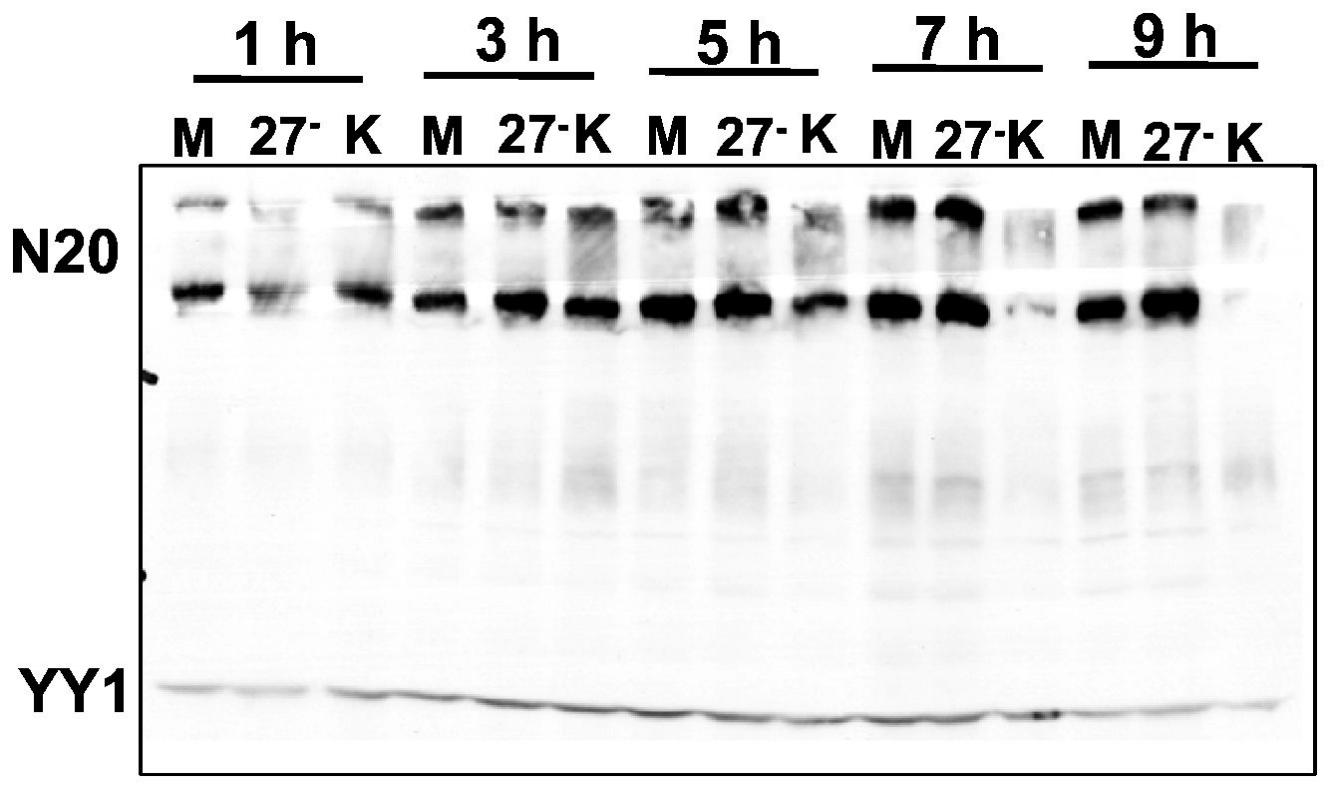
Total RNAP II levels decrease during WT HSV-1 KOS infection. HeLa cells were either mock infected or infected with WT HSV-1 KOS or with an ICP27 null mutant, 27-GFP in which the ICP27 coding sequence was replaced with GFP. Cells lysates were prepared at 1, 3, 5, 7 and 9 h after infection and separated on a 5-15% linear gradient SDS-polyacrylamide gel. Western blots were probed with polyclonal antibody N20, which detects all forms of RNAP II The cellular protein YY1 served as the loading control.

We showed previously that during wild type HSV-1 KOS infection, levels of serine-2 phosphorylated RNAP II were significantly reduced, and in fact levels of total RNAP II, both hyperphosphorylated and hypophosphorylated were decreased by around 5 h after infection when HSV-1 transcription and DNA replication are highly active [24]. This decrease in total RNAP II levels can be seen in Figure 1, which shows a western blot analysis of whole cell lysates from cells that were mock infected or were infected with HSV-1 ICP27 null mutant 27-GFP or WT KOS for 1, 3, 5, 7 and 9 h. Blots were probed with antibody N20, a polyclonal antibody that recognizes an epitope in the N-terminus of the large subunit of RNAP II and therefore, which recognizes all forms of RNAP II (Table 1). By 5 h after infection, there was a decrease in both hyperphosphorylated and hypophosphorylated RNAP II in KOS infected cells and this decrease was even more pronounced at 7 and 9 h after infection when viral transcription is highly robust (Figure 1). In contrast, as we reported previously, when ICP27 was not expressed during infection, there was little decrease in RNAP II levels (Figure 1). We inferred that this is because ICP27 is required to relocalize RNAP II to viral transcription-replication sites [19,24] and during 27-GFP infection, viral transcription of early and late genes is greatly reduced [24,27]. These results led us to postulate that during highly robust viral transcription in WT KOS infected cells, elongating transcription complexes might collide or pile up and stall. We further showed that RNAP II becomes ubiquitinated during KOS infection and that proteasome inhibitors were able to prevent the decrease in RNAP II levels, indicating that stalled RNAP II complexes were likely being degraded by the proteasome [24]. Thus, the decrease in RNAP II levels and particularly in phosphoserine-2 levels may be attributed to proteasomal degradation of stalled elongating RNAP II transcription complexes during highly active transcription in WT HSV-1 infections. 

**Table 1:** Specificity of antibodies against RNA polymerase II.

**Antibody**	**RNAP II form**	**Epitope**
ARNA3 (monoclonal)	All forms, unphosphorylated, initiating and elongating	N-terminal epitope in large subunit of RNAP II holoenzyme
N-20 (rabbit polyclonal)	All forms, unphosphorylated, initiating and elongating	N-terminal epitope in the large subunit of RNAP II holoenzyme
H14 (monoclonal)	RNAP II-initiating complex	Ser-5-phosphorylated CTD
H5 (monoclonal)	RNAP II-elongating complex	Ser-2-phosphorylated CTD

### Effects of DRB and FVP on RNAP II and HSV-1 protein levels

**Figure 2:**
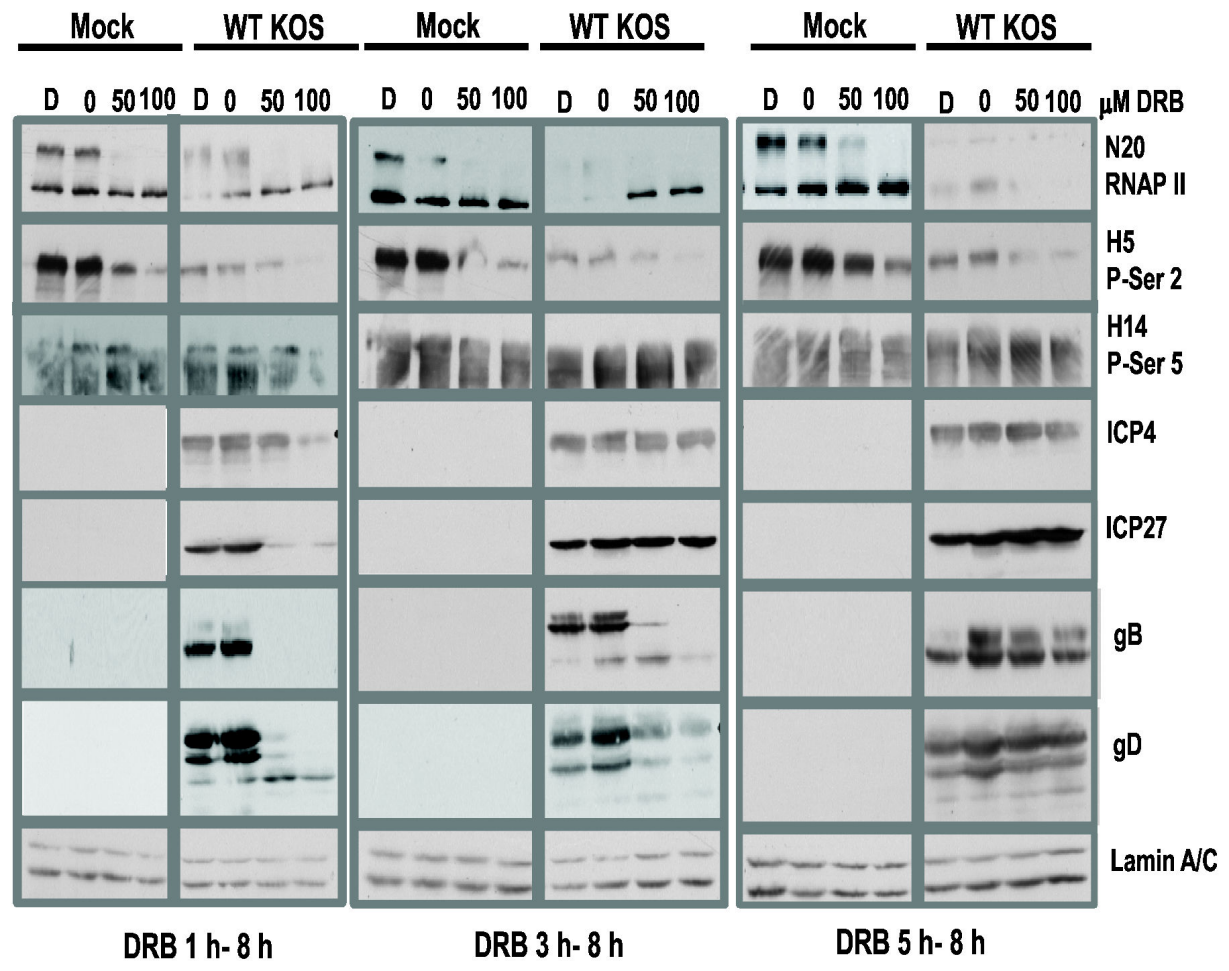
RNAP II CTD phosphoserine-2 levels decreased with increasing amounts of DRB. HeLa cells were mock infected or infected with WT HSV-1 KOS at an MOI of 10. DRB stocks were prepared in Dimethyl formamide. At 1, 3 and 5 h after infection, Dimethyl formamide alone (D) or increasing amounts of DRB from 0 to 100µµM were added to cell monolayers. At 8 h after infection, whole cell lysates were prepared and separated on 5-15% linear gradient SDS-polyacrylamide gels. Western blots were probed with polyclonal antibody N20, which detects all forms of RNAP II; monoclonal antibody H5, which detects phosphoserine-2, and monoclonal antibody H14, which detects phosphoserine-5. For HSV-1 proteins, antibody P1011 was used to detect IE protein ICP4, P1119 for ICP27, P1123 for gB and P1103 for gD. Lamin A/C served as a sample loading control.

Because the phosphoserine-2 form of RNAP II CTD is decreased during HSV-1 KOS infection as we [24] and Fraser and Rice showed [25], we wanted to address the importance of the phosphoserine-2 form to viral gene expression during HSV-1 infection. In mammalian cells serine-2 phosphorylation of RNAP II CTD is required for transcription elongation as well as for RNA processing [6,7,9,14,15,17]. RNAP II CTD is phosphorylated on serine-2 by kinase cdk9, which acts in conjunction with cyclin 1 in mammalian cells and the complex is referred to as P-TEFb for positive transcription elongation factor [2,3]. DRB is an adenosine analogue that is a specific inhibitor of cdk9. Although DRB can also affect the activity of other kinases, its affinity for cdk9 at IC 50 µM ranges from 3 fold greater than its affinity for cdk7 to more than 10 fold higher for cdk9 compared to CKII and PKA [37]. Analysis of the crystal structure of cdk9 in complex with DRB showed that DRB chlorine atoms form halogen bonds with oxygen in the cdk9 hinge region and that is the basis for its specificity [5]. We first determined the effect of different concentrations of DRB on the phosphorylated forms of RNAP II in mock infected cells compared to HSV-1 KOS infected cells. Increasing concentrations of DRB were added at 1, 3 and 5 h after infection and whole cell lysates were isolated at 8 h after infection and western blot analysis was performed (Figure 2). In mock infected cells, addition of 50 or 100 µM DRB resulted in a loss of the more slowly migrating hyperphosphorylated form of RNAP II detected by antibody N20 with a shift to the faster migrating hypophosphorylated form (Figure 2). In HSV-1 KOS infected cells, the results were more complex. When DRB was added at 1 and 3 h after infection, hypophosphorylated RNAP II was seen with antibody N20 at both concentrations of DRB with little of the hyperphosphorylated form visible (Figure 2). In the samples to which Dimethyl formamide alone or no DRB were added, there was a pronounced decrease in both forms of RNAP II in KOS infected cells. We interpret this result to mean that in the absence of the inhibitor, robust HSV-1 transcription transpired during the 8 h infection, which would result in stalled elongation complexes and proteasomal degradation of RNAP II as described earlier and shown in Figure 1. When DRB was added at 5 h after infection, very low levels of RNAP II were detected in the presence or absence of DRB. This finding suggests that by 5 h after infection, proteasomal degradation of RNAP II is already occurring as shown in Figure 1, and addition of DRB would further reduce the hyperphosphorylated form by blocking cdk9 activity (Figure 2). 

In looking specifically at the phosphoserine-2 form as detected with antibody H5, the addition of 100 µM DRB was more effective than 50 µM in reducing serine-2 phosphorylation in mock infected cells, as were longer incubation times with DRB present (Figure 2). In HSV-1 KOS infected cells, there was a significant reduction in phosphoserine-2 RNAP II in the Dimethyl formamide alone or no DRB samples. This in accordance with previous results that demonstrated that the serine-2 form of RNAP II is degraded during HSV-1 infection, which proceeded for 8 h with no drugs in these control samples [24]. Treatment with 100 µM DRB reduced phosphoserine-2 to an even greater extent compared to 50 µM DRB. That DRB was specific for cdk9 at the concentrations used in these studies was shown by probing the blots with antibody H14, which recognizes phophoserine-5 (Table 1). Serine-5 is phosphorylated by cdk7. Serine-5 levels were largely unaffected by DRB in mock or KOS infected cells regardless of the DRB concentration or time of addition (Figure 2) These results demonstrate that the addition of 100 µM DRB is more effective in reducing serine-2 phosphorylation of RNAP II CTD by cdk9.

To determine what effect reducing phosphoserine-2 levels would have on HSV-1 immediate early (IE) and late protein levels, we analyzed the expression of IE proteins ICP4 and ICP27 and late proteins glycoprotein B (gB) and glycoprotein D (gD) in the presence and absence of DRB. ICP4 levels were only slightly decreased when 100 µM DRB was added 1 h after infection, and were not appreciably affected by 50 µM DRB or when DRB was present for shorter times during infection (Figure 2). A similar result for ICP4 was reported by Durand and Roizman [23] who monitored ICP4 expression in cells treated with 50 µM DRB for 8 h beginning 2 h after infection. ICP27 levels were significantly reduced by the addition of 50 and 100 µM DRB when added 1 h after infection but were unaffected when DRB was added at later times (Figure 2). Levels of late proteins gB and gD were significantly decreased when DRB was added at 1 and 3 h after infection but were not visibly affected by DRB addition at 5 h. 

**Figure 3:**
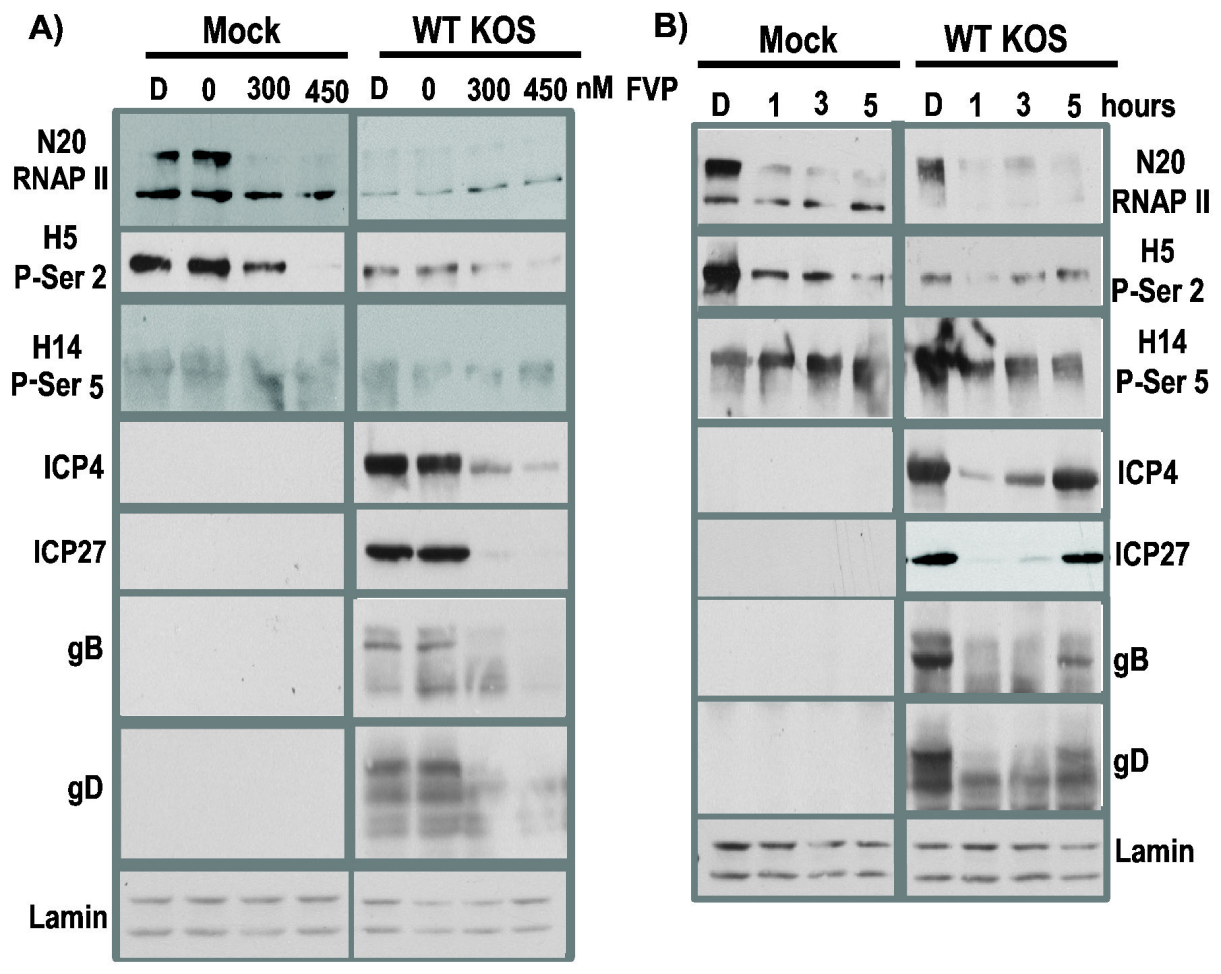
Addition of FVP reduced RNAP II CTD phosphoserine-2 levels and levels of HSV-1 IE proteins ICP4 and ICP27 and late proteins gB and gD were also reduced. A) HeLa cells were mock infected or infected with WT HSV-1 KOS at MOI of 10. . Flavopiridol (FVP) stocks were prepared in DMSO. At 1 h after infection, DMSO alone or increasing amounts of FVP from 0 to 450 nM were added to cell monolayers. At 8 h after infection, whole cell lysates were prepared and fractionated on 5-15% gradient SDS-polyacrylamide gels. Western blots were probed with N20, H5 and H14 as indicated. Lamin A/C served as the loading control. B) DMSO alone was added at 0 h after infection or FVP (450 nM) was added at 1, 3 or 5 h after infection as indicated. Whole cell lysates were prepared at 8 h. Western blots were probed with anti-RNAP II antibody N20 or phosphoserine-2 antibody H5 or phosphoserine-5 antibody H14. HSV-1 protein ICP4 was detected using antibody P1101; ICP27 was detected with antibody P1119; gB was detected with antibody P1103 and gD was detected with antibody 1123. Lamin A/C served as a loading control.

Next, we probed the importance of RNAP II phosphoserne-2 during HSV-1 infection by using another inhibitor of cdk9, namely FVP, which has been used extensively to study the role of P-TEFb on transcription elongation [3,37–42]. FVP binds to the ATP site of cdk9 through ATP-like hydrogen bond interactions that induce structural changes in cdk9 [43]. FVP inhibits cdk9 activity specifically. First we tested different concentrations of FVP (Figure 3). DMSO alone or increasing concentrations of FVP were added to mock and HSV-1 KOS infected cells at 1 h after infection and whole cell lysates were prepared at 8 h after infection. In mock infected cells,, 300 and 450 nM concentrations of FVP were effective in shifting hyperphosphorylated RNAP II to the hypophosphorylated form as detected by antibody N20 (Figure 3A). A similar result was seen in KOS infected cells (Figure 3A). Here again, in the absence of FVP, total levels of RNAP II detected by N20 were greatly reduced in accord with proteasomal degradation of RNAP II during the 8 h HSV-1 infection. A concentration of 450 nM FVP was more effective in reducing phosphoserine-2 RNAP II as detected by antibody H5 for both mock and KOS infected cells (Figure 3A), and no significant effect was seen in phosphoserine-5 levels detected by antibody H14. In contrast to the results that we observed with DRB, addition of FVP at either concentration at 1 h after infection diminished ICP4, ICP27, gB and gD levels (Figure 3A). To determine how the time of treatment with FVP would affect RNAP II and HSV-1 proteins, 450 nM FVP was added to mock and KOS infected cells at 1, 3 and 5 h after infection and cell lysates were harvested at 8 h after infection (Figure 3B). Total RNAP II levels as detected by N20 were significantly decreased in KOS infected cells, reflecting both a reduction in hyperphosphorylated RNAP II by addition of FVP early in infection and proteasomal degradation of elongating complexes when FVP was added at later times of infection. Phosphoserine-2 was significantly and specifically decreased by FVP as seen in the H5 panels for both mock and KOS infected cells compared to the H14 samples, showing that serine-5 phosphorylation was generally unaffected. The time of addition of FVP was important for HSV-1 protein expression. Addition of FVP at 1 and 3 h after infection significantly affected ICP4 and ICP27 protein levels, whereas there was a marginal effect when FVP was added at 5 h (Figure 3B). Late proteins gB and gD were more adversely affected when FVP was added at 1 and 3 h after infection but addition at 5 h also resulted in lower levels of these late proteins. 

The results with DRB and FVP demonstrate that RNAP II phosphoserine-2 levels were specifically reduced in both mock and KOS infected cells and that expression of IE proteins ICP4 and ICP27 were affected more adversely when FVP was added at earlier times. Levels of late proteins gB and gD were reduced when DRB and FVP were added at earlier times after infection but later addition of FVP still decreased gB and gD levels.

**Figure 4:**
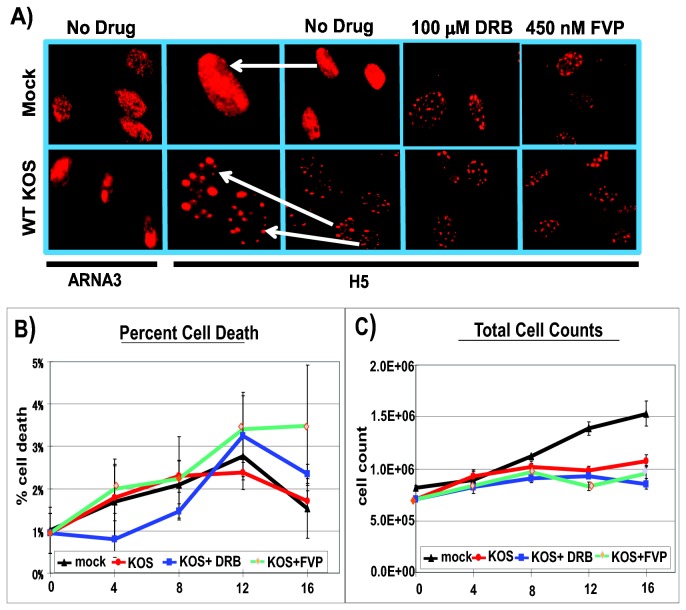
DRB and FVP altered the staining pattern of H5 antibody. A) RSF cells were either mock infected or infected with WT HSV-1 KOS at an MOI of 10. Cells were either left untreated (no drug) or were treated with 100 µM DRB or 450 nM FVP as indicated. Cells were fixed at 8 h and stained with RNAP II antibody ARNA3, which recognizes all forms of RNAP II or phosphoserine-2 antibody H5. For the H5 no drug panels, cells from mock and KOS samples have been shown at higher magnification to show the nuclear staining patterns and are identified by white arrows. B-C) To account for cell toxicity, HeLa cells were either mock infected, or infected with HSV-1 KOS at an MOI of 1 for up to 16 hours as indicated. Infected cells were either untreated or treated with 100 µM DRB or 450 nM FVP. Cells were collected at 4 h intervals, stained with Trypan Blue vital dye, and counted in triplicate using a hemocytometer to estimate B) percent cell death and C) total cell counts.

In previous studies we showed that in HSV-1 KOS infected cells, when phosphoserine-2 levels were reduced, immunofluorescent staining with antibody H5 was seen in speckled structures in the nucleus instead of diffuse staining throughout the nucleus as seen in mock infected cells [24,27]. A similar result was also reported by Fraser and Rice [25]. The reason for this change in the staining pattern is because it was reported that antibody H5 cross-reacts with a phospho-epitope in SR splicing proteins under conditions in which phosphoserine-2 RNAP II is less abundant compared to highly abundant SR proteins [44]. This occurs during HSV-1 infection because of proteasomal degradation of elongating RNAP II complexes as described earlier [24] and because HSV-1 ICP22 can also cause a decrease in the phosphoserine-2 form [26]. Therefore, H5 staining is a convenient method to detect loss of phosphoserine-2 RNAP II. When mock and KOS infected cells were stained with antibody ARNA3, which recognizes an epitope in the N-terminus, and thus all forms of RNAP II (Table 1), a diffuse nuclear staining was seen in mock and KOS infected cells (Figure 4A). When antibody H5 was used, this diffuse nuclear staining pattern was seen in mock infected cells that were not treated with inhibitors, whereas in KOS infected cells, a speckled staining pattern was seen, consistent with H5 recognizing SR proteins in splicing speckles as reported previously [24,25,27]. Upon treatment with DRB or FVP, both mock and KOS infected cells showed the speckled staining pattern with antibody H5, consistent with a loss in the serine-2 phosphorylated form of RNAP II.

To assess the cytotoxicity of the inhibitors, percent cell death was estimated by trypan blue staining at 4 h intervals in mock vs. KOS infected cells that were or were not treated with DRB or FVP (Figure 4B). Total cell counts were also estimated at 4 h intervals (Figure 4C). A marked increase in cell death was not observed in the presence of the inhibitors.

### DRB and FVP reduced RNA synthesis in mock and HSV-1 infected cells

**Figure 5:**
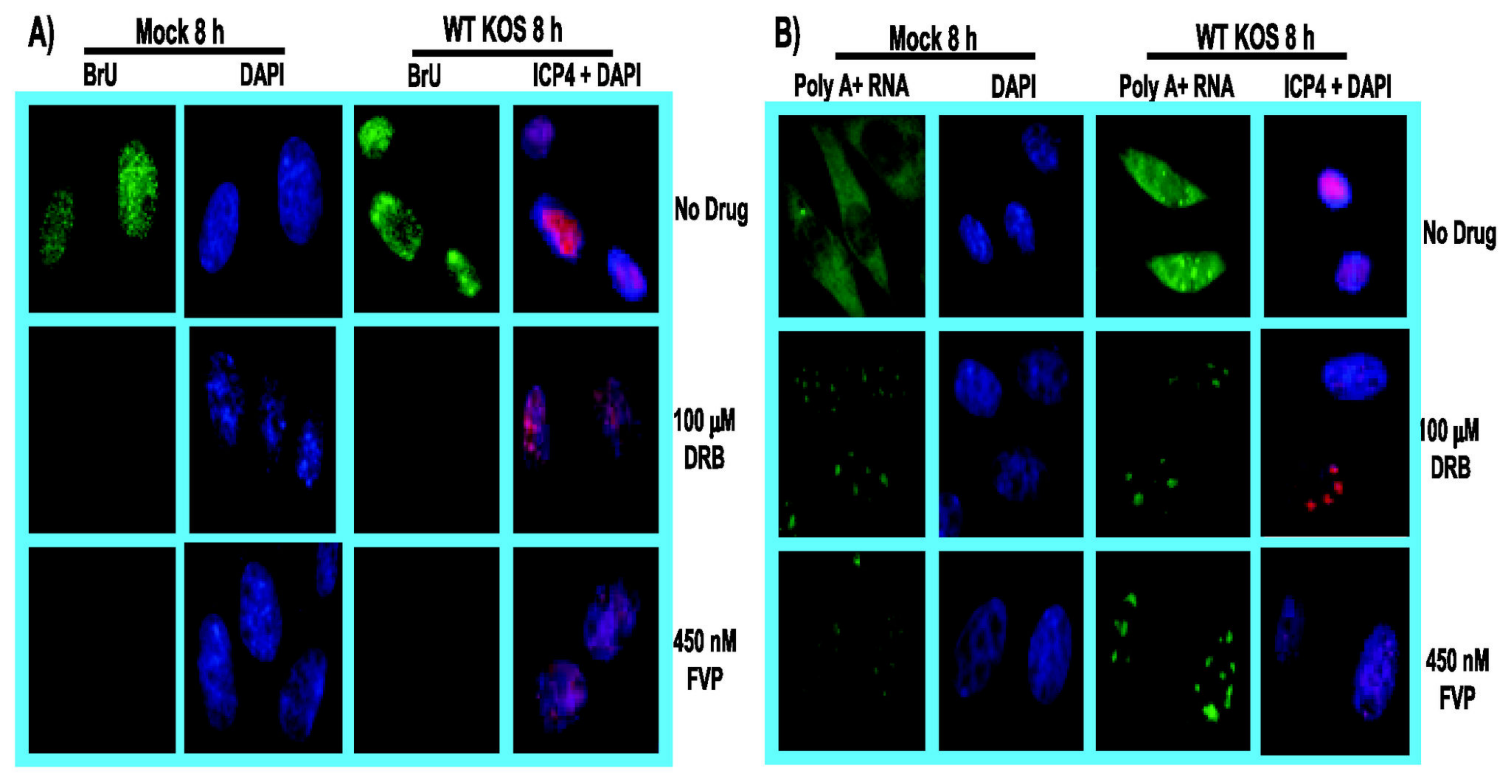
DRB and FVP reduced RNA synthesis in mock and HSV-1-infected cells. RSF cells were mock infected or infected with WT HSV-1 KOS at MOI of 10. At 1 h after infection, cells were treated with DRB (100 µM) or FVP (450 nM). A) Bromouridine (BrU) was added to the media (4 mM) at 7.5 h to label nascent RNA. Cells were fixed at 8 h after infection and stained with anti-BrU antibody (green). Mock infected cells were stained with DAPI to mark nuclei. KOS-infected cells were stained with DAPI and with anti-ICP4 antibody as a marker of infection. B) Cells were fixed at 8 h after infection and in situ hybridization with a biotinylated oligo-dT probe was performed to detect poly(A+) RNA. Mock infected cells were stained with DAPI and KOS infected cells were stained with DAPI and anti-ICP4 antibody as an infection marker. All images were captured on a Zeiss Axiovert 200M microscope at 100X magnification.

 To determine the effect of adding cdk9 inhibitors on RNA synthesis and accumulation, mock and HSV-1 KOS infected cells were treated with DRB or FVP beginning 1 h after infection (Figure 5). To monitor newly synthesized RNA, 5-Bromouridine (BrU) was added for 30 min at 7.5 h after infection to pulse label newly transcribed, nascent RNA. Cells were subsequently fixed and stained with anti-BrU antibody. In the absence of inhibitors, BrU incorporation was observed in mock and KOS infected cells (Figure 5A). However, BrU incorporation was not detected in mock or KOS infected cells treated with DRB or FVP (Figure 5A), suggesting that RNAP II transcription was curtailed by these cdk9 inhibitors. To assess the effect of these inhibitors on accumulation of poly(A)+ mRNA, in situ hybridization was performed with an oligo-dT probe (Figure 5B). In both mock and KOS infected cells (infected cells were marked by staining for ICP4), in the absence of inhibitors, poly(A)+ RNA was seen throughout the nucleus (marked by DAPI staining) and the cytoplasm (Figure 5B). However, after addition of DRB or FVP, poly(A)+ RNA was observed to be concentrated in large speckles in the nucleus and was barely detectable in the cytoplasm. These results indicate that inhibition of cdk9 by DRB and FVP negatively affected RNA synthesis and accumulation in both mock and HSV-1 infected cells, indicating that the phosphoserine-2 form of RNAP II is required for transcription during HSV-1 infection just as it is required in uninfected cells.

### The effects of DRB and FVP on HSV-1 infection can be reversed

**Figure 6:**
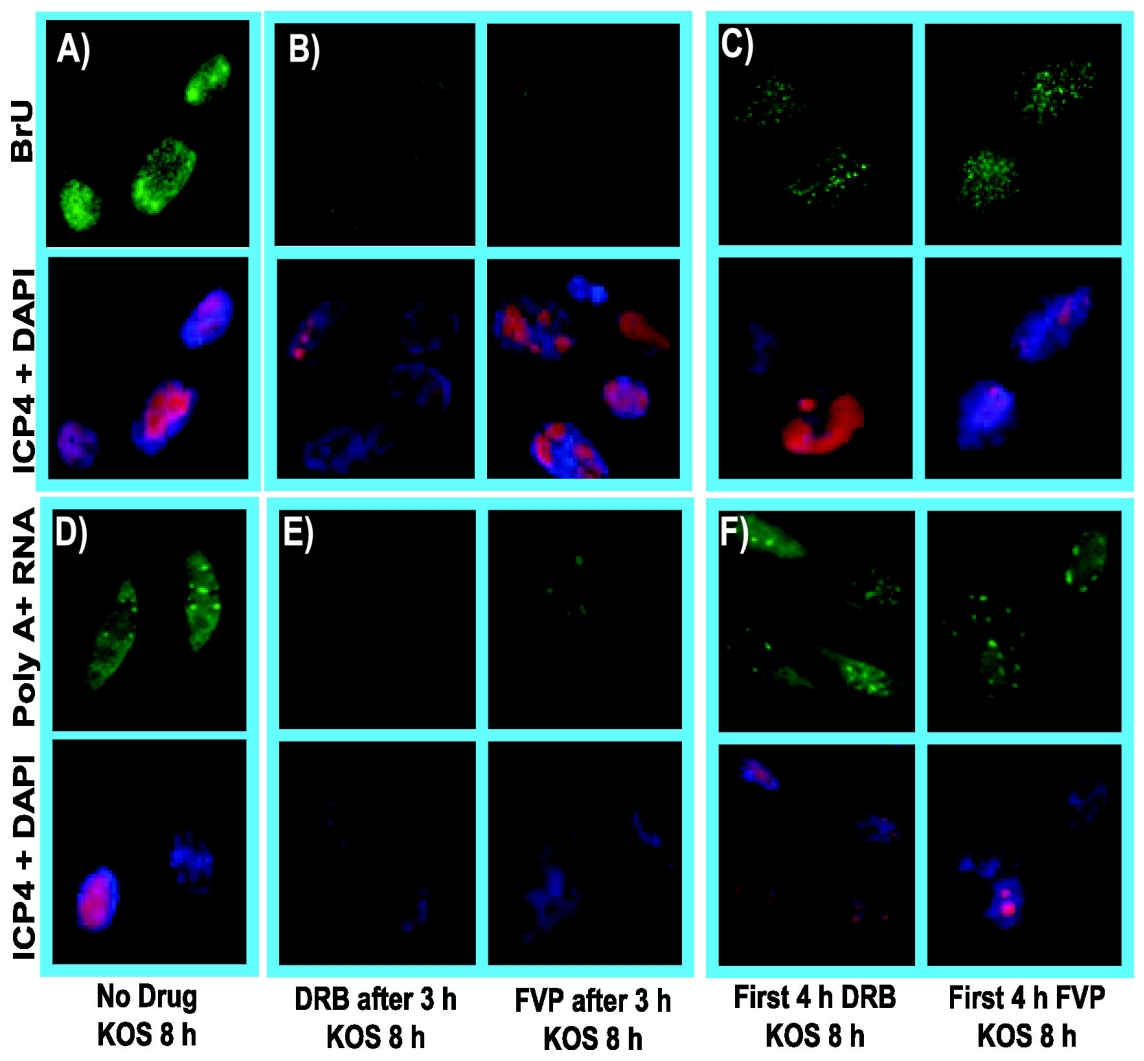
The inhibitory effects of cdk9 inhibiters DRB and FVP on RNA synthesis during HSV-1 infection could be reversed after removing the drugs. RSF cells were infected with HSV-1 KOS at an MOI of 10. A, D) Infected cells were left untreated. B, E) Infected cells were treated with 100 µM DRB or 450 nM FVP at 3 h after infection. C,F) Infected cells were treated with 100 µM DRB or 450 nM FVP as indicated for the first 4 h of infection after which, cells were washed and drug-free medium was added. A-C). BrU was added to the media at 7.5 h after infection to label nascent RNA. Cells were fixed at 8 h and stained with anti-BrU and anti-ICP4 antibodies and with DAPI. D-F). Cells were fixed at 8 h after infection and in situ hybridization was performed with a biotinylated oligo dT-probe, which was subsequently detected by FITC-conjugated streptavidin. ICP4 staining served as an infection marker and DAPI staining marked nuclei. All images were captured on a Zeiss Axiovert 200M microscope at 100X magnification.

To determine if the effects of these inhibitors could be reversed during HSV-1 infection, DRB or FVP were added for the first 4 h of infection and then washed away and drug free medium was added for an additional 4 h. These samples were compared to HSV-1 KOS infected cells that were left untreated and to KOS infected cells to which DRB or FVP were added at 3 h after infection and remained in the medium until 8 h when all cells were fixed and stained (Figure 6). In the absence of drugs, BrU incorporation was seen (Figure 6A) and poly(A)+ RNA was distributed throughout the nucleus and cytoplasm (Figure 6D). When DRB or FVP were added at 3 h and remained in the medium until 8 h, there was no detectable incorporation of BrU during a 30 min pulse at 7.5 h (Figure 6B) and poly(A)+ RNA was observed in large speckles in the nucleus and was barely detectable in the cytoplasm (Figure 6E). In the case of DRB or FVP addition from the start of infection until 4 h, after which the drugs were washed away and drug free medium was added for an additional 4 h, BrU incorporation was again observed during a 30 min pulse at 7.5 h (Figure 6C). Poly(A)+ RNA was also observed in the nucleus and cytoplasm (Figure 6F), although for both BrU incorporation and poly(A)+ RNA hybridization, the fluorescence signals were less intense than those observed in the absence of drugs. Nevertheless, it appears that the effects of DRB and FVP on RNA synthesis and accumulation were reversible.

**Figure 7:**
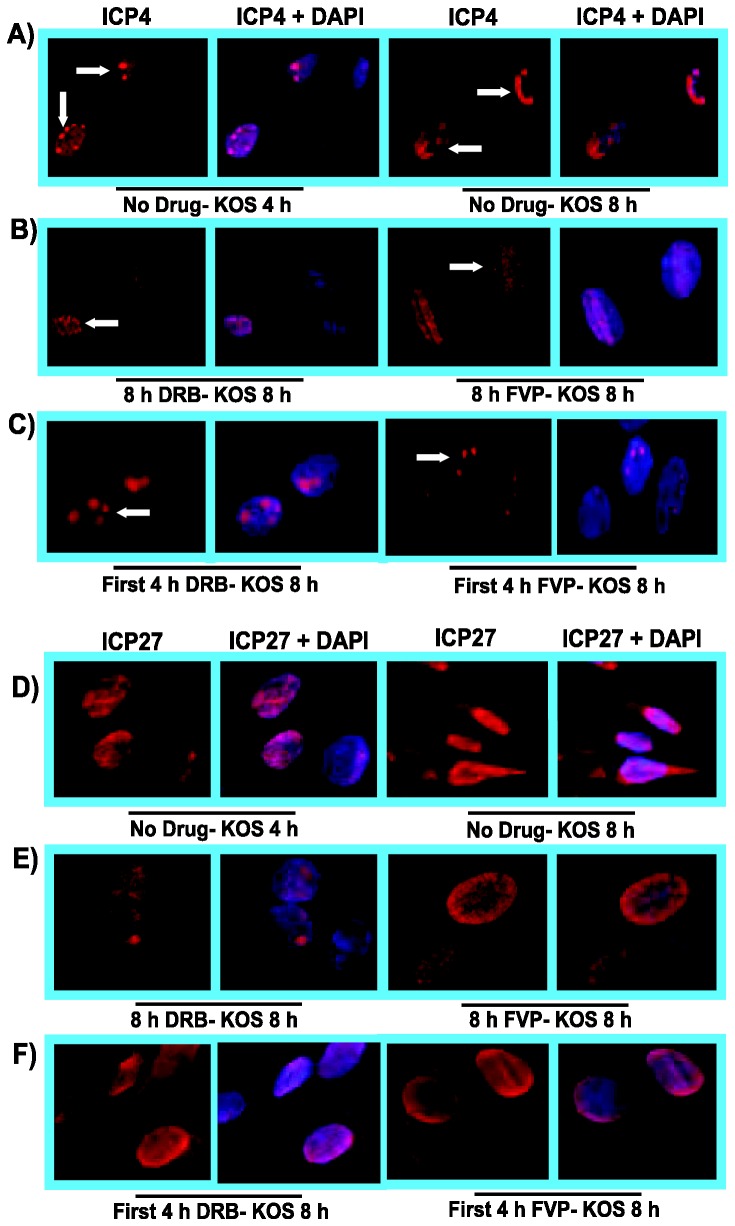
DRB and FVP hindered HSV-1 transcription-replication compartment formation but pre-replicative compartments were apparent after drug removal. RSF cells were infected with WT KOS at an MOI of 10. A,D) Infected cells were untreated and were fixed at 4 h and 8 h as indicated. B,E) Cells were treated with 100 µM DRB or 450 nM FVP as indicated at the beginning of infection and were fixed at 8 h. C,F) DRB or FVP were added at the start of infection and were removed at 4 h and cells were incubated in drug-free medium for an additional 4 h. Infected cells were fixed at 8 h. A,B,C). Cells were stained with anti-ICP4 antibody to monitor viral transcription-replication compartment formation and DAPI to mark nuclei. D,E,F). Infected cells were stained with anti-ICP27 antibody to monitor ICP27 sub-cellular localization and DAPI to mark nuclei. Images were captured on a Zeiss Axiovert 200M microscope at 100X magnification.

 As a further test of whether the effects of these inhibitors on HSV-1 infection were reversible, we looked at the localization of viral IE proteins ICP4 and ICP27 in the presence of DRB or FVP and after the removal of the drugs. ICP4 is a transcriptional activator [45] that serves as a marker for viral transcription-replication compartments [46,47], which first form as pre-replicative sites at 4 h after infection (Figure 7A) and which form full blown replication compartments by 8 h after infection (Figure 7A). When DRB or FVP were present throughout infection, ICP4 was diffusely distributed in the nucleus (Figure 7B), however, when DRB and FVP were removed after 4 h and infected cells were incubated an additional 4 h, small replication compartments and pre-replicative sites were seen (Figure 7C) indicating that viral replication could resume. During HSV-1 infection, ICP27 is nuclear at early times but it begins shuttling between the nucleus and cytoplasm at later times in its role as a viral mRNA export factor [34,35,48–50] (Figure 7D). In the presence of DRB or FVP, ICP27 remained in the nucleus throughout infection (Figure 7E) but when the drugs were removed at 4 h, ICP27 was detected in the cytoplasm 4 h later (Figure 7F), again indicating that the inhibitory effects of DRB and FVP on viral infection were reversible when these inhibitors were removed. 

### A global reduction in HSV-1 mRNA expression in the presence of DRB and FVP

**Figure 8:**
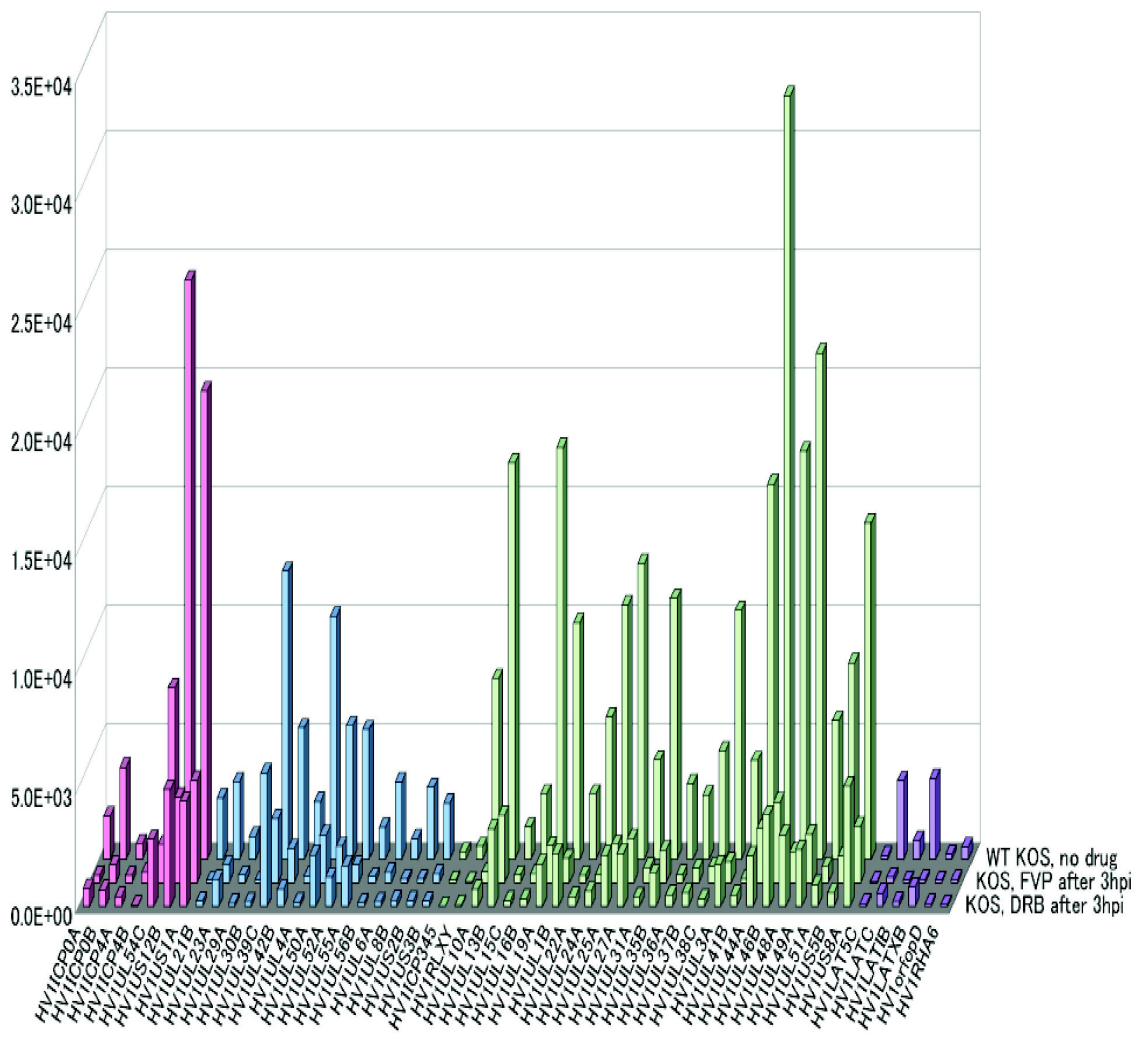
A global reduction in HSV-1 mRNA expression was seen in the presence of DRB and FVP. HeLa cells were infected with HSV-1 KOS at an MOI of 10 and were either left untreated (no drug) or were treated with 100 µM DRB or 450 nM FVP starting at 3 h after infection. Total RNA was isolated at 8 h. Poly(A+) RNA was selected and reverse transcribed and the cDNA from each fraction was hybridized to HSV-1-specific microarray chips and quantified by using Array Vision software. The graph shown displays the averages of three independent experiments in which each transcript was represented three times on the array. The y axis represents the intensity of the light scattering signal and the x axis represents individual HSV-1 transcripts. Red bars represent immediate-early transcripts; early transcripts are in blue; late transcripts are in green, and LAT transcripts are in violet.

 To determine the effect of cdk9 inhibitors DRB and FVP on individual HSV-1 transcript levels, total poly(A)+ RNA was isolated at 8 h after infection from KOS infected cells to which no inhibitors were added and from KOS infected cells to which DRB or FVP were added at 3 h after infection. Following reverse transcription of selected poly(A)+ RNA, cDNA from each fraction was hybridized to HSV-1 specific microarrays as described previously [27,35]. A global reduction was seen in transcripts from all kinetic classes, ie, early and late when DRB or FVP were added at 3 h after infection (Figure 8). We conclude that inhibition of cdk9 by DRB and FVP inhibited HSV-1 transcription.

**Figure 9:**
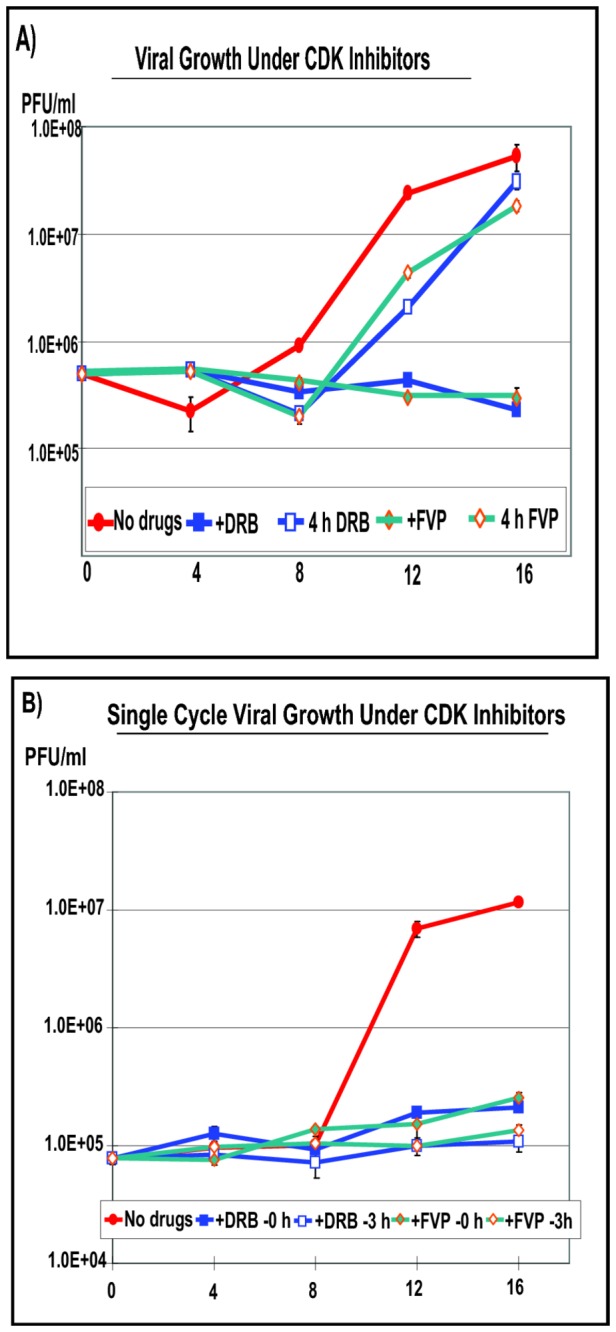
Viral replication was greatly reduced in the presence of DRB and FVP but viral replication resumed when the drugs were removed after 4 h. HeLa cells were infected with HSV-1 KOS at MOI of 1. A) Infected cells were either untreated (no drugs) or DRB (100 µM) or FVP (450 nM) were added at the time of infection for the duration of infection, or DRB and FVP were present for the first 4 h and then were removed and cells were incubated in drug-free medium for the remainder of the time, as indicated. Samples were harvested at 0, 4, 8, 12 and 16 h after infection and virus titers were determined by plaque assays. B) HeLa cells were infected with KOS at an MOI of 1 and were untreated (no drugs) or treated with DRB or FVP starting at 0 h or 3 h as indicated and the drugs were present for the duration of the experiment. Samples were harvested at 0, 4, 8, 12 and 16 h as described in panel A. The experiments were performed in triplicate and error bars are shown.

 To determine the effect of these inhibitors on viral replication, one step growth curves were performed. In the presence of DRB or FVP added at the time of infection, viral replication was completely inhibited (Figure 9A). In contrast, when DRB or FVP were present for the first 4 h after infection but were removed thereafter, viral replication resumed by 12 h (Figure 9A). A single cycle growth curve was also performed in which DRB and FVP were added at the time of infection or at 3 h. Viral titers were similarly reduced in the presence of these inhibitors added either at the start of infection or 3 h later (Figure 9B). These results indicate that inhibition of cdk9 prevented viral replication but removal of the inhibitors allowed viral replication to resume, which further supports the conclusion that the phosphoserine-2 form of RNAP II is required for HSV-1 replication.

### Effects of a dominant negative cdk9 mutant or over expression of HEXIM1 on nascent RNA synthesis

**Figure 10:**
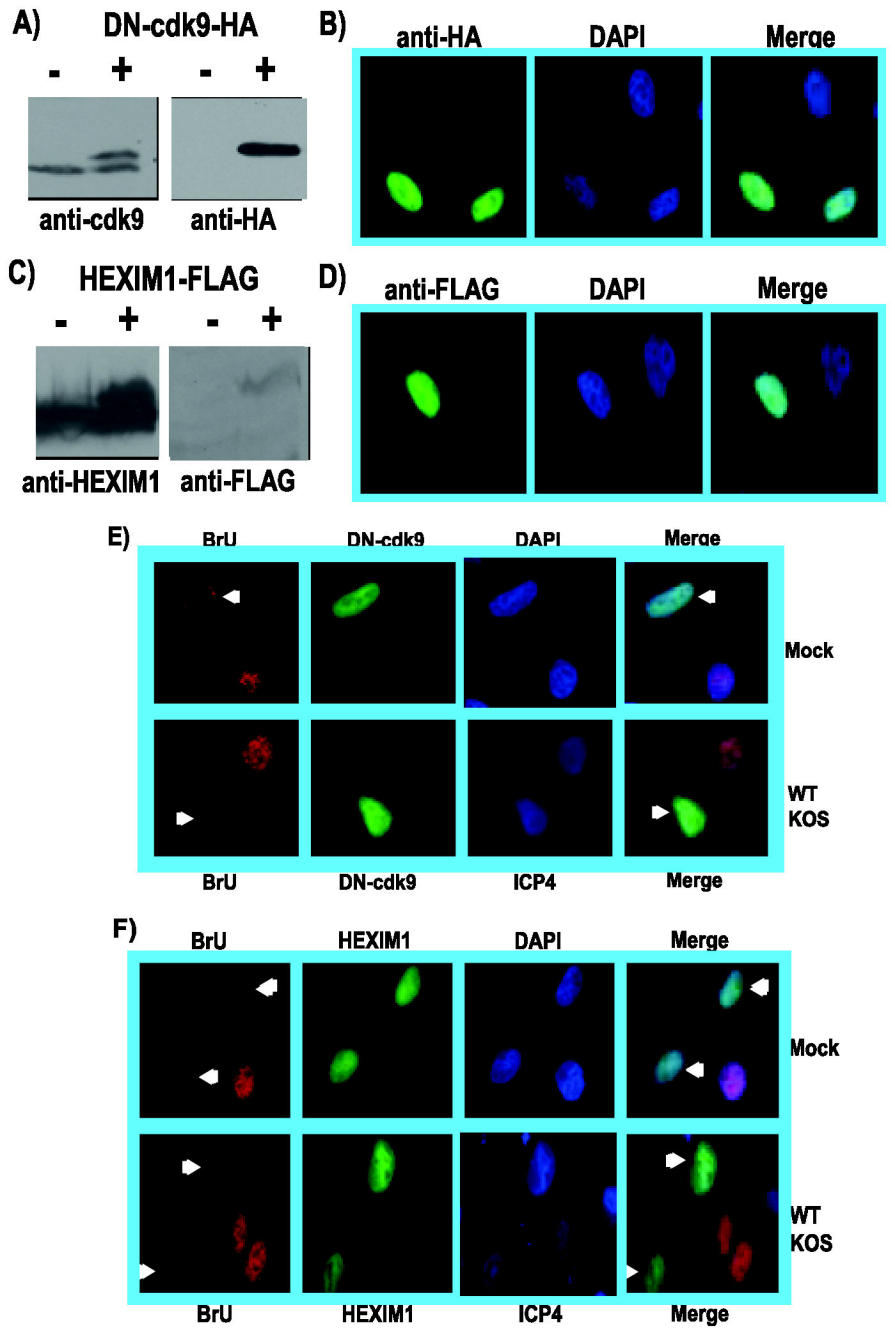
Expression of a dominant-negative kinase-dead cdk9 mutant or the cdk9 negative-regulator HEXIM1 inhibited nascent RNA synthesis. A) HeLa cells were either transfected with empty vector or with 2 µg of DN-cdk9-HA DNA, which expresses an HA-tagged kinase-dead cdk9 dominant negative mutant. After 24 h, whole cell lysates were prepared and fractionated by SDS-PAGE. Western blots were probed with anti-cdk9 antibody (left panel) or anti-HA antibody (right panel). B) HeLa cells transfected with DN-cdk9-HA were fixed 24 h after transfection and stained with anti-HA antibody to visualize DN-cdk9-HA and DAPI to mark nuclei. C) HeLa cells were transfected with empty vector or with plasmid DNA expressing FLAG-tagged HEXIM1. Whole cell lysates were prepared as in panel A and Western blots were probed with anti-HEXIM1 antibody (left) and anti-FLAG (right). D) Indirect immunofluorescence was performed on HeLa cells transfected with HEXIM1-FLAG. Anti-FLAG antibody was used to visualize HEXIM1-FLAG and DAPI staining marked the nuclei. E) HeLa cells were transfected with DN-cdk9-HA for 24 h and were subsequently mock infected or infected with HSV-1 KOS. Bromouridine (BrU) was added at 7.5 h after infection for 30 min, at which time cells were fixed and stained with anti-BrU antibody to visualize newly transcribed RNA and anti-HA antibody to visualize DN-cdk9-HA. DAPI staining in the upper mock panels marked nuclei and staining with anti-ICP4 antibody in the lower KOS panels was used as a marker for infection. White arrows point to cells expressing DN-cdk9-HA in the BrU and merged panels. F) HeLa cells transfected with HEXIM1-FLAG for 24 h were mock infected or infected with HSV-1 KOS and BrU was added at 7.5 h after infection for 30 min. Cells were fixed at 8 h and stained with anti-BrU to detect newly transcribed RNA and anti-FLAG antibody to detect HEXIM1-FLAG. DAPI was used to mark nuclei in the upper mock panels and anti-ICP4 antibody was used in the lower KOS panels as a marker for infection. White arrows point to cells expressing HEXIM1-FLAG. All images were captured on a Zeiss Axiovert 200M microscope under 100X magnification.

 As an alternative to chemical inhibitors, we also targeted cdk9 activity through expression of a dominant negative kinase-dead mutant and through over expression of HEXIM1. It has been shown that expression of mutant DN-cdk9, a kinase dead mutant [31],, in HeLa cells results in the preferential inhibition of phosphorylation of RNAP II serine-2 without affecting phosphorylation of serine-5 [4,31]. Here we transfected HeLa cells with empty vector or a plasmid expressing DN-cdk9 tagged with an HA-epitope-tag. Western blot analysis was performed on cell lysates 24 h after transfection to confirm DN-cdk9-HA expression (Figure 10A) and immunofluorescent staining with anti-HA antibody was also performed (Figure 10B). DN-cdk9-HA was found to be expressed at levels similar to endogenous cdk9 (Figure 10A). To determine the effect of DN-cdk9 expression on nascent RNA synthesis, HeLa cells transfected with DN-cdk9-HA for 24 h were either mock infected or were infected with HSV-1 KOS for 8 h. At 7.5 h after infection, BrU was added for 30 min and cells were fixed and stained. BrU incorporation was greatly reduced in the cell that expressed DN-cdk9-HA compared to the cell that did not in both mock and KOS infected samples (Figure 10E). About 50 fields of cells were assessed for BrU incorporation. Greater than 90% of the cells that were stained with HA antibody showed little to no incorporation of BrU, whereas cells that did not express DN-cdk9-HA did show staining with BrU antibody. This indicates that DN-cdk9 expression interfered with cdk9 activity and this impaired nascent RNA synthesis in both mock and KOS infected cells.

 HEXIM1 (hexamethylene bisacetamide-inducible protein 1) is a negative regulator of P-TEFb, which consists of cdk9 and cyclin 1 [32,40,51,52]. The PYNT motif of HEXIM1 is required to inhibit the kinase activity of cdk9, although the mechanism has not been elucidated. HEXIM1 binds 7SK non coding RNA and P-TEFb associates with HEXIM1 forming an inactive P-TEFb complex [40,53]. We transfected HeLa cells with FLAG-tagged HEXIM1 or empty vector and analyzed cell lysates 24 h later by western blot to monitor HEXIM1-FLAG expression (Figure 10C). HEXIM1-FLAG was expressed at levels similar to endogenous HEXIM1 levels. HEXIM1-FLAG expression was also confirmed by immunofluorescent staining (Figure 10D). To determine the effect of HEXIM1-FLAG expression on BrU incorporation, HeLa cells that were transfected with HEXIM1-FLAG for 24 h were either mock infected or were infected with HSV-1 KOS and BrU was added for 30 min 7.5 h after infection. Fixed cells were stained with anti-BrU and anti-FLAG antibodies. BrU incorporation was not detectable in the cells expressing HEXIM1-FLAG in both mock and KOS infected cells. Again, about 50 fields of cells were assessed and greater than 90% of the cells that were stained with anti-FLAG antibody displayed little to no BrU staining, whereas, cells that did not stain with anti-FLAG, indicating HEXIM1-FLAG was not being expressed, did stain with anti-BrU antibody. This indicates that over expression of HEXIM1 inhibited the kinase activity of cdk9, which in turn resulted in an inhibition of nascent RNA synthesis in mock and KOS infected cells. 

We conclude that inhibition of cdk9 by specific inhibitors DRB, FVP, DN-cdk9 or HEXIM1 results in decreased viral transcription, indicating that the phosphoserine-2 form of RNAP II is required during HSV-1 infection, just as it is required in uninfected cells.

## Discussion

 During HSV-1 infection there is a decrease in the phosphoserine-2 form of RNAP II CTD at later times of infection when transcription of viral genes is very robust, and which results in part from proteasomal degradation of stalled elongating transcription complexes [24,27]. It has also been reported that ICP22 contributes to the loss of the serine-2 form by an as yet undefined mechanism [26]. Further, it has been shown that ICP22 and cdk9 are present in a complex with the CTD of RNAP II [22,23] and ICP22 and viral protein kinases U_L_13 and U_S_3 can cause a mobility shift change in the phosphorylation pattern of RNAP II resulting in an intermediately phosphorylated form detectable by CTD-specific antibody 8WG16 [22,54]. This antibody does not recognize phosphoserine-2 because the serine at position 2 of the CTD is part of its epitope recognition, which appears to be altered or occluded upon phosphorylation [55]. The actual CTD phosphorylated sites of the intermediately phosphorylated form have not been clarified. The sites of phosphorylation on the CTD by viral kinases U_L_13 and U_S_3 have also not been determined. It has also been proposed that the serine-2 phosphorylated form of RNAP II, which is required for transcription elongation, may not be required during HSV-1 infection. Here, we specifically targeted cellular kinase cdk9, which phosphorylates serine-2 and found that inhibition of cdk9 activity impaired HSV-1 transcription globally, indicating that the phosphoserine-2 form of RNAP II CTD is required for HSV-1 transcription as it is for cellular transcription. 

 The decrease in the phosphoserine-2 form of RNAP II during HSV-1 infection is unusual and is not seen with several other viruses, which have been shown to require cdk9 for their gene expression. Specifically, transcription elongation appears to be a critical regulator for viral latent infection. When the HIV-1 genome is integrated, transcription through the long terminal repeat promoter is regulated at the level of transcription elongation [38,39,41]. The HIV-1 transactivator Tat recruits P-TEFb to stalled RNAP II complexes that have initiated at the LTR, and phosphorylation by cdk9 results in processivity of HIV-1 transcription [39]. HTLV-1 protein Tax also complexes with P-TEFb through cyclin T1 to regulate the balance between active and inactive P-TEFb complexes [56]. During Kaposi’s sarcoma-associated herpesvirus (KSHV) latent infection, it has been shown that RNAP II transcription complexes are paused at the promoters of KSHV lytic genes OriLyt1, K5, K6 and K7 by the negative elongation factor NELF[11], which results in hyperphosphorylation of serine-5 and hypophosphorylation of serine-2. If these genes are induced to be expressed by the viral lytic activator Rta, the viral lytic phase ensues resulting in viral replication and cell death. Thus, negative regulation of transcription elongation of these lytic genes keeps KSHV in the latent state [11]. Similarly, in another gamma herpesvirus, Epstein-Barr virus (EBV), the viral latent state is also maintained by NELF-DSIF [57]. Specifically, the viral C promoter (Cp) that drives a viral pre-mRNA of about 120 kb, which is differentially spliced to produce several EBV products that are required for immortalization, displays high levels of promoter-proximal stalled RNAP II. P-TEFb is recruited to Cp by the cellular bromodomain protein Brd4 and inhibitor studies with DRB showed that P-TEFb is required for Cp transcription [57].

 The beta herpesvirus, Human Cytomegalovirus (HCMV) recruits cdk9 to viral nuclear replication compartments during lytic infection and this recruitment results in hyperphosphorylation of RNAP II CTD [58–61]. HCMV proteins IE2 86 [60] and UL69 [61] have been shown to be required for the recruitment of cyclin T1 and cdk9 to viral replication compartments. Thus, cdk9 appears to be important for HCMV lytic replication. 

The studies that we have described here would also indicate that cdk9 is important for HSV-1 replication. One consequence of highly active HSV-1 transcription may be an increasing number of stalled transcription elongation complexes in crowded areas of the genome and clearing these by proteasomal degradation results in lower levels of phosphoserine-2 RNAP II from the stalled elongating complexes. How ICP22 causes a decrease in serine-2 levels is still a bit of an enigma. Durand and Roizman [22,23] showed that cdk9 interacts with ICP22. They further showed that cdk9 and ICP22 colocalize with RNAP II. Inhibition of cdk9 with 50 µM DRB and with cdk9 siRNA resulted in decreased expression of several HSV-1 late genes that were previously shown to be regulated by ICP22. In a recent study using an *in vivo* transient expression reporter system [62], ICP22 was coimmunoprecipitated with P-TEFb in accord with the results of Durand and Roizman. Further using ChIP assays, Guo et al. showed that ICP22 blocked the recruitment of P-TEFb to viral promoters, which inhibited transcription of these promoters [62], In this system, VP16 recruited P-TEFb to promoters and counteracted transcriptional repression by ICP22 [62] Because this was a transient expression system, it is not clear if ICP22 would similarly repress viral promoters by preventing P-TEFb recruitment during viral infection. Another possibility might be that ICP22, like HIV Tat recruits inactive P-TEFb in complex with 7SK snRNA and HEXIM1 to viral promoters and another factor, perhaps VP16, can cause its disassociation into active P-TEFb. Thus, HSV-1 might have a complex regulatory mechanism to insure that transcription of early and late viral genes can occur during very active times after infection when both DNA replication and transcription are proceeding robustly. It will be necessary to parse and define many details of this regulation during HSV-1 infection and to determine how ICP22 affects cdk9 activity, all of which are beyond the scope of this study. In conclusion, we showed here that cdk9 activity is required for HSV-1 transcription and replication.
